# Trends of Non-Traumatic Lower-Extremity Amputation and Type 2 Diabetes: Spain, 2001–2019

**DOI:** 10.3390/jcm11051246

**Published:** 2022-02-25

**Authors:** Ana Lopez-de-Andres, Rodrigo Jimenez-Garcia, Valentin Hernandez-Barrera, Javier de Miguel-Diez, Jose M. de Miguel-Yanes, Ricardo Omaña-Palanco, David Carabantes-Alarcon

**Affiliations:** 1 Department of Public Health & Maternal and Child Health, Faculty of Medicine, Universidad Complutense de Madrid, IdISSC, 28007 Madrid, Spain; anailo04@ucm.es (A.L.-d.-A.); romana@ucm.es (R.O.-P.); dcaraban@ucm.es (D.C.-A.); 2 Preventive Medicine and Public Health Teaching and Research Unit, Health Sciences Faculty, Universidad Rey Juan Carlos, Alcorcón, 28032 Madrid, Spain; valentin.hernandez@urjc.es; 3 Respiratory Department, Hospital General Universitario Gregorio Marañón, Instituto de Investigación Sanitaria Gregorio Marañón (IiSGM), Universidad Complutense de Madrid, 28007 Madrid, Spain; javier.miguel@salud.madrid.org; 4 Internal Medicine Department, Hospital General Universitario Gregorio Marañón, Instituto de Investigación Sanitaria Gregorio Marañón (IiSGM), Universidad Complutense de Madrid, 28007 Madrid, Spain; josemaria.demiguel@salud.madrid.org

**Keywords:** amputation: lower extremity, diabetes, hospitalization, mortality, sex differences

## Abstract

(1) Background: To examine trends in the incidence (2001–2019), clinical characteristics and in-hospital outcomes following major and minor non-traumatic lower-extremity amputations (LEAs) among people with type 2 diabetes mellitus (T2DM) in Spain, assessing possible sex differences. (2) Methods: Retrospective cohort study using data from the Spanish National Hospital Discharge Database. Joinpoint regression was used to estimate incidence trends, and multivariable logistic regression to estimate factors associated with in-hospital mortality (IHM). (3) Results: LEA was coded in 129,059 patients with T2DM (27.16% in women). Minor LEAs accounted for 59.72% of amputations, and major LEAs comprised 40.28%. The adjusted incidences of minor and major LEAs were higher in men than in women (IRR 3.51; 95%CI 3.46–3.57 and IRR 1.98; 95%CI 1.94–2.01, respectively). In women, joinpoint regression showed that age-adjusted incidence of minor LEAs remained stable over time, and for major LEAs, it decreased from 2006 to 2019. In men, incidences of minor and major LEAs decreased significantly from 2004 to 2019. In-hospital mortality (IHM) increased with age and the presence of comorbidity, such as heart failure (OR 5.11; 95%CI 4.61–5.68, for minor LEAs and OR 2.91; 95%CI 2.71–3.13 for major LEAs). Being a woman was associated with higher IHM after minor and major LEA (OR 1.3; 95%CI 1.17–1.44 and OR 1.18; 95%CI 1.11–1.26, respectively). (4) Conclusions: Our data showed major sex differences indicating decreasing and increasing LEA trends among men and women, respectively; furthermore, women presented significantly higher IHM after minor and major LEA procedures than men.

## 1. Introduction

Lower-extremity amputations (LEAs) are a frequent complication suffered by people with type 2 diabetes mellitus (T2DM) and imply loss of quality of life and higher risk of short-term mortality [1]. Rates of LEAs among people with T2DM are a good indicator of the quality of diabetes care [1]. In Spain, the number of T2DM-related major LEAs have decreased between 2001 and 2012 [2,3]. The pooled analysis of studies conducted in 21 countries suggested that the improvement in the quality of diabetes health care has contributed to a positive evolution, with a decline in major LEA rates over the last years [4]. However, for minor LEAs, no significant reductions or even small increments have been reported [4,5].

Sex differences have been described previously in regard to incidence rates and short- and long-term outcomes after LEAs among people with T2DM [2,3,6,7,8,9]. Several studies suggest that men have higher incidence rates than women [2,3,6,7,8], but being a woman is associated with more frequent surgical site infections and a higher risk of dying in the hospital following LEAs [3,9].

Reliable data obtained from national hospital discharge databases can be useful to identify sex differences regarding the trends overtime and the hospital outcomes among people with T2DM. The results of this investigations could help health resource planning and healthcare providers. 

Using discharge data from an entire country from 2001 to 2019, the objectives of our investigation were: (i) to examine sex differences in the incidence overtime of major and minor LEAs among people with T2DM; (ii) to assess changes overtime in the clinical profile and in-hospital outcomes in women and men with T2DM who underwent a major or minor LEA; (iii) to identify among people with T2DM the role of sex and other variables in the IHM according to LEA types.

## 2. Materials and Methods

### 2.1. Design, Setting and Participants

We conducted a retrospective cohort study. The data source was the records from year 2001 to 2019 collected by the Spanish National Hospital Discharge Database (RAE-CMBD, Registro de Actividad de Atención Especializada. Conjunto Mínimo Básico de Datos, Registry of Specialized Health Care Activities. Minimum Basic Data Set). This registry used the International Classification of Disease, 9th Revision, Clinical Modification (ICD-9-CM) from 2001 to 2015 and the 10th Revision (ICD-10) from 2016 through the end of 2019. 

The RAE-CMBD only includes patients who stay in a hospital room for at least 24 h. Therefore, patients seen at the emergency department or outpatient setting were not analyzed in this investigation. Details on RAE-CMBD are available online [10]. 

For study purposes we excluded data from year 2016, because in this year the RAE-CMBD began the conversion from ICD-9 to ICD-10, and according to the Ministry of Health, some degree of under-codification may exist [11,12]. 

The study population was stratified by sex and included patients with T2DM aged ≥18 years with a procedure code for LEAs in their discharge records. The ICD-9-CM and ICD-10 codes used to identify the study population are shown in Table S1. We defined as a minor amputation any LEA distal to the ankle joint, and a major amputation as any LEA through or proximal to the ankle joint, as described in a previous study [3]. 

We excluded patients with a diagnosis code of T1DM and all those with traumatic LEAs (Table S1).

For patients who had more than one LEA conducted during their hospitalization, we selected, for study purposes, the higher-level LEA.

### 2.2. Study Variables

The main study variables were the incidence of major and minor LEAs among men and women with T2DM, clinical characteristic, length of hospital stay (LOHS) and in- hospital mortality (IHM). 

Data from the Spanish National Health Surveys (NHS) conducted in the two year periods 2001/02, 2003/04, 2006/07, 2009/10, 2011/12, 2014/15, 2016/17 and 2019/20, and data from the Di@bet.es Study were used to obtain the denominators for incidence rates calculations [13,14]. The number of people with diabetes for the years when the NHS was not conducted (2005, 2008, 2013 and 2018) were inferred, assuming that the change in prevalence was linear.

Patient-level variables analyzed included age, sex and clinical conditions prior to hospital admission or first diagnosed during the hospitalization. The clinical conditions analyzed included those of the Charlson Comorbidity Index (CCI) as described elsewhere [15,16]. The CCI was analyzed as a continuous variable. Furthermore, we specifically described and analyzed the following diseases and conditions (Table S1): obesity, peripheral vascular disease, ischemic heart disease, chronic kidney disease, hypertension, stroke, heart failure and lipid metabolism disease due to their high prevalence and importance among people with T2DM.

### 2.3. Statistical Analysis

For the study purposes, the period from 2001 to 2019 was divided into six two-year periods: 2001–2003; 2004–2006; 2007–2009; 2010–2012; 2013–2015 and 2017–2019. 

Mean with standard deviation (SD) or median with interquartile range (IQR); are provided to describe quantitative variables and total frequencies and percentage for categorical variables.

T Student-test, Mann–Whitney test and Chi-square test were conducted for bivariate comparisons. To assess time trends for categorical variables, the Cochran-Armitage tests for trend was used.

We used Joinpoint Regression Program (version 4.0.4) to identify the periods in which trend changes in age-adjusted rates for minor and major LEAs among women and men with T2DM occurred, as well as to estimate the annual percentage of change (APC) in each of the periods delimited by the points of change [17]. 

Differences according to sex in the incidence rates for LEAs were estimated using age-adjusted Poisson regression. 

To assess the independent effect of sex and other study variables on the IHM after LEAs among people with T2DM, we conducted multivariable logistic regression. 

The statistical analysis was conducted using Stata version 17 (Stata, College Station, TX, USA), and significance was set at *p* < 0.05 (two-sided).

### 2.4. Ethical Aspects

As the RAE-CMBD is a totally anonymized administrative database in accordance with Spanish legislation, it did not require ethics committee approval. The database can be freely accessed upon request [18].

## 3. Results

In our study, we identified a total of 129,059 LEAs in patients aged ≥18 years with T2DM in Spain between 2001 and 2019. The proportion of women was 27.16% (35,054 non-traumatic amputations). Minor LEA procedures were identified in 77,082 admissions (women accounted for 22.4%) and 51,977 hospitalizations were identified as major LEA procedures (34.16% women).

### 3.1. Sex Differences in Incidence, Clinical Characteristics, and Hospital Outcomes for People with T2DM who Underwent Minor LEA Procedure

The total incidence of minor LEA procedures was higher (*p* < 0.001) among men with T2DM (335.23 per 100,000 men with T2DM) than among women with T2DM (89.11 per 100,000 women with T2DM), resulting in an adjusted IRR of 3.51 (95%CI 3.46–3.57).

In patients who underwent minor LEAs, women were significantly older than men (72.33 vs. 67.32 years; *p* < 0.001) and had less coexisting medical conditions (mean CCI 1.05 vs. 1.14; *p* < 0.001). Specifically, women had lower prevalence of peripheral vascular disease, ischemic heart disease and lipid metabolism disorder, However, hypertension (51.13% vs. 30.33%; *p* < 0.001) and heart failure (8.45% vs. 7.26%; *p* < 0.001) were more frequently codified among women. The median LOHS was 15 days in both women, and men. The crude IHM rate was higher in women than in men with T2DM (3.6% vs. 2.15%; *p* < 0.001).

Over time, patient age increased significantly only in men with T2DM who underwent minor LEAs (*p* < 0.001). We found a significant increase over time in comorbidity according to the mean CCI in both sexes (0.97 and 0.84, respectively, in 2001/03 vs. 1.25 and 1.19, respectively, in 2017/19; all *p* < 0.001). Specifically, an increase was observed in the prevalence of ischemic heart disease, chronic kidney disease, hypertension and heart failure in both groups. In men and women, the prevalence of lipid metabolism disorders was over four times higher in 2017/19 (10.42% and 11.12%, respectively, in 2001/03, vs. 45.94% and 45.95%, respectively, in 2017/19; *p* < 0.001). However, the prevalence of peripheral vascular disease decreased slightly over time in women (53.06% in 2001/03 vs. 51.51%, in 2017/19; *p* < 0.010).

In both groups, median LOHS and IHM decreased significantly over time. In men with T2DM, IHM was 2.69% in the period 2001/03, decreasing significantly to 1.87% in 2017/19 (*p* < 0.001). The same trend was seen in women (*p* < 0.001), with IHM falling from 3.98% to 2.74% (*p* < 0.001)., as can been seen in Table 1.

### 3.2. Sex Differences in Incidence, Clinical Characteristics, and Hospital Outcomes for People with T2DM Who Underwent Major LEA Procedure

We found that the total incidence of major LEAs was higher among men with T2DM (191.85 per 100,000 men with T2DM) than among women (91.51 per 100,000 women with T2DM). The Poisson regression model yielded an adjusted IRR for men of 1.98 (95% CI 1.94–2.01).

When we compared men and women with T2DM who underwent major LEAs, we found that women were older than men (77.34 years vs. 71.61 years; *p* < 0.001) and had less comorbidity than men (mean CCI 1.33 vs. 1.48; *p* < 0.001). Women had lower prevalence of peripheral vascular disease, ischemic heart disease, chronic kidney disease and lipid metabolism disorder; however, women had higher prevalence of hypertension (52.47% vs. 44.28%; *p* < 0.001). The median LOHS was 15 days and 17 days in women and men, respectively. The crude IHM rate was higher in women than in men with T2DM (11.53% vs. 8.8%; *p* < 0.001), as can been seen in Table 2.

We found that the incidence of major LEA procedure increased slightly from 173.44 per 100,000 men with T2DM in 2001/03 to 178.82 in 2017/19 (*p* = 0.004). However, in women, the total incidence decreased significantly from 101.12 in 2001/03 to 66.93 per 100,000 women with T2DM in 2017/19 (*p* < 0.001), as can been seen in Table 2.

Age remained stable over time among men with T2DM, however in women with T2DM it increased significantly (*p* < 0.001). The CCI increased significantly over time in both men and women (all *p* < 0.001). As can been seen in Table 2, the prevalence of peripheral vascular disease, chronic kidney disease, hypertension and heart failure increased over time in both men and women with T2DM. As we found for minor LEAs, the prevalence of lipid metabolism disorder in men and women with T2DM was over 5-times higher in 2017/19 (9.2% and 9.18%, respectively, in 2001/03 vs. 45.93% and 45.27%, respectively, in 2017/19; *p* < 0.001).

As can been seen in Table 2, median LOHS decreased significantly over time in both men and women groups. In men with T2DM, IHM was 9.86% in 2001/03, decreasing significantly to 8% in 2017/19 (*p* = 0.002). The same trend was seen in women, with IHM falling from 11.11% to 10.14% (*p* = 0.019).

### 3.3. Joinpoint Analysis for Women and Men with T2DM Who Underwent Minor and Major Non-Traumatic Lower-Extremity Amputations

**Figure 1:**
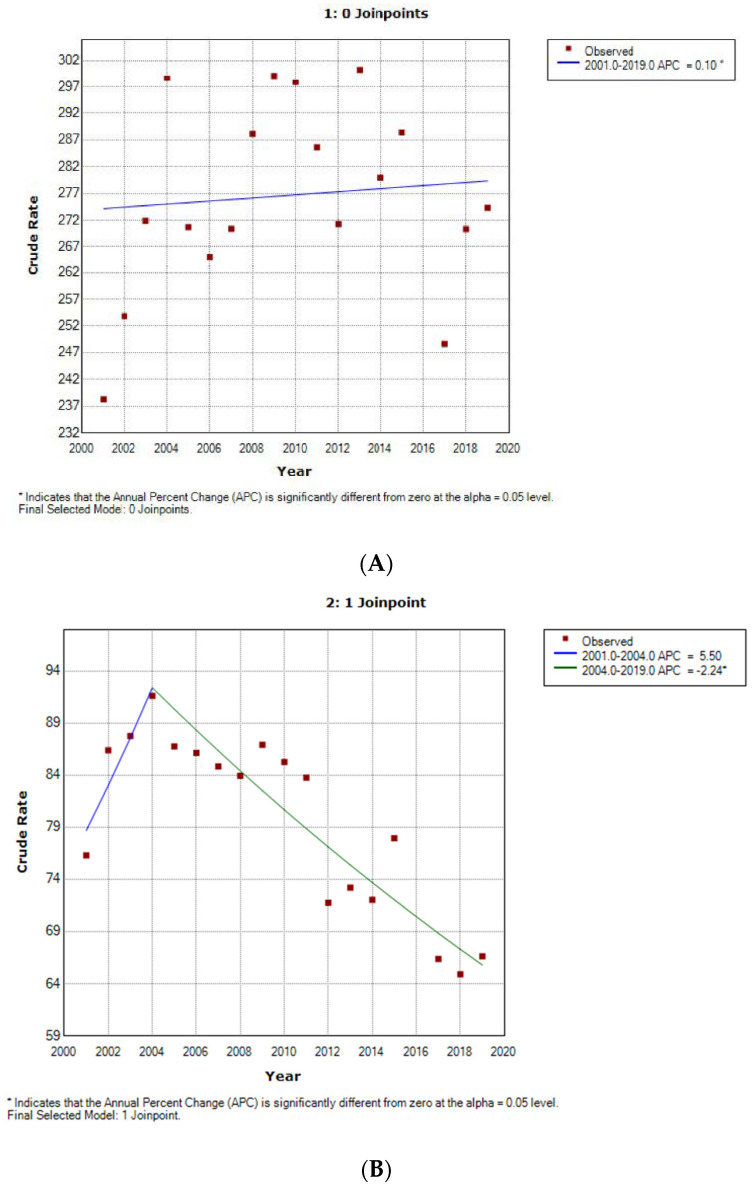
Joinpoint analysis for minor non-traumatic lower-extremity amputations among people with type 2 diabetes mellitus according to sex. (**A**) Women, (**B**) Men.

According to the results of the joinpoint analysis, we found that the incidence of age-adjusted minor LEA procedures in women with T2DM remained stable from 2001 to 2019 (Figure 1A). Incidence decreased significantly in men by 2.24% per year from 2004 to 2019, with a non-significant increase before 2004 (Figure 1B).

**Figure 2:**
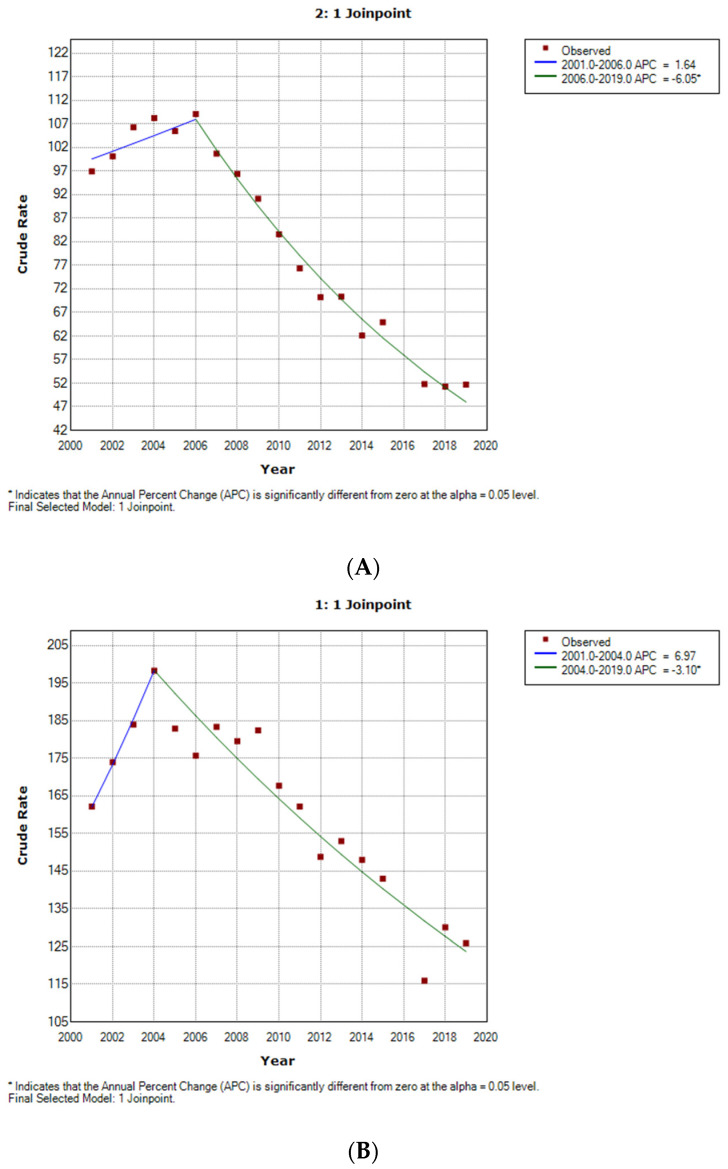
Joinpoint analysis for major non-traumatic lower-extremity amputations among people with type 2 diabetes mellitus according to sex. (**A**) Women, (**B**) Men.

The joinpoint analysis showed that among women with T2DM who underwent major LEAs, the incidence rate showed no significant change from 2001 to 2006, and then decreased significantly by 6.05% per year from 2006 to 2019 (Figure 2A). Men showed a similar trend, with the decline starting in year 2004 with an APC of 3.10% until 2019 (Figure 2B).

### 3.4. In-Hospital Mortality among People with T2DM after Non-Traumatic Lower-Extremity Amputations According to Sex

As can been seen in Table 3, in men and women after minor and major LEAs, IHM was significantly higher in the older age groups and in those with comorbidities, such as peripheral vascular disease, ischemic heart disease, chronic kidney disease, hypertension, stroke and lipid metabolism disorder. Furthermore, men and women with heart failure had the highest risk of dying after LEAs (for minor LEAs, 10.26% and 14.03%, respectively, and for major LEAs, 19.81% and 25.82%, respectively). 

In men and women with T2DM, after minor and major LEAs, IHM was highest when the hospital admission occurred in the 2001–2003 period (Table 3). 

### 3.5. Predictors of Dying in the Hospital among People with T2DM after Non-Traumatic Lower-Extremity Amputations

As can been seen in Table 4, using the entire database including people with T2DM, after multivariable adjustment, the risk of dying in hospital increased with age, ischemic heart disease, chronic kidney disease and stroke among patients who underwent minor and major LEAs. Furthermore, heart failure was the condition that most increased the risk of dying for minor LEAs (OR 5.11; 95%CI 4.61–5.68) and major LEAs (OR 2.91; 95%CI 2.71–3.13). 

The time-trend analysis showed a significant decrease in IHM from 2001/03 to 2017/19 in people with T2DM who underwent minor and major LEAs (Table 4). 

Finally, higher IHM was associated with being a woman in people with T2DM after minor and major LEAs (OR 1.30; 95%CI 1.17–1.44 and OR 1.18; 95%CI 1.11–1.26, respectively). This means that, after adjusting for possible confounders, women with T2DM who underwent a major or a minor LEAs had a 30% and an 18% higher risk, respectively, of dying during their hospitalization than men with T2DM who underwent these same procedures.

The variables independently associated with IHM among men and women with T2DM who underwent a major or a minor LEA are shown in Table S2. For all these study groups, the risk of dying rose along with age and with the presence of concomitant chronic conditions. Specifically, the highest ORs were found for heart failure, followed by stroke, chronic kidney disease and ischemic heart disease. Improvement overtime was found for men and women who underwent minor LEAs, but only among men with T2DM for major LEAs (Table S2).

## 4. Discussion

This population-based observational study showed differences according to sex among patients hospitalized with T2DM following a LEA procedure. Incidence rates of minor and major LEAs in men with T2DM were higher than in women with T2DM between years 2001 and 2019. After multivariable adjustment, women with T2DM had a 30% and 18% higher risk of dying in the hospital following minor and major LEAs, respectively, than men with T2DM.

The results of our study agree with others, finding an increasing male predominance in minor and major LEAs [3,19,20,21]. Differences in the prevalence of cardiovascular risk factors, such as tobacco use, the protective effect of estrogen for women, and biological factors of diabetic foot ulcer, peripheral neuropathy and peripheral vascular disease are among the causes proposed to explain these higher rates among T2DM men [21,22,23,24,25,26].

During admission for minor and major LEAs, we found that, before and after multivariable adjustment, women with T2DM had higher IHM than men with T2DM. Furthermore, among women with T2DM who underwent major LEAs, no change in IHM was found overtime. There are no conclusive results so far regarding the association of sex with IHM following LEAs [3,27,28,29]. Wong et al reported that long-term mortality was higher in women, and they explained this result as pertaining to greater age and low physical activity levels among women patients [27]. Gurney et al. [28] reported no sex-difference in postoperative mortality (90 days), even when women who underwent an LEA had a similar distribution of baseline risk factors for postoperative complications. However, among people undergoing vascular surgery, women are usually under-represented, and this can result in lower crude mortality rates. This is misleading, since women have been found to have poorer outcomes after vascular surgery than men [29]. 

The mechanisms that could explain the higher risk of vascular complications in women with diabetes when compared to men are multifactorial [30,31,32,33,34,35]. Besides hormonal and genetic factors, social factors, such as socio-economic status and inequalities in access to care and treatments have been demonstrated to negatively affect women [30,35]. Indeed, in Spain, IHM after major cardiovascular events has been found to be higher among T2DM women than men [36]. 

According to joinpoint analysis, trends in incidence decreased significantly for major LEAs in men and women with T2DM, and for minor LEAs only among men. Most, but not all studies, have reported declining rates of LEAs over the last years [4,37,38,39,40]. In Canada, Hussain et al concluded that from 2005 to 2016, the rates of LEAs in people with diabetes have declined slower than other cardiovascular events [41]. 

As described in the literature, our results show an increasing ratio of minor-to-major LEAs among men and women with T2DM. It has been suggested that this may be due to a more aggressive treatment of peripheral arterial disease, with earlier minor LEAs to prevent major amputations [42].

Non-traumatic LEAs have been associated with high IHM [2,3]. As previously described in the literature, the presence of comorbidities, such as chronic kidney disease and coronary artery disease, increased mortality [43,44]. In the present study age, ischemic heart disease, chronic kidney disease, stroke and heart failure were found to be significant risk factors that increase IHM. Belonging to the oldest age group (≥80 years old) increases the IHM by 15.6 and 3.15 times in minor and major LEAs, respectively, when compared to the youngest group (18–49 years). Many studies have shown that age is a risk factor for mortality [45,46]. We found that the most significant risk factor for IHM among men and women with T2DM who had undergone minor and major LEAs was the presence of heart failure. Similarly, other authors have reported that heart failure is an important risk factor that increases mortality after LEAs [47].

When the entire study population was analyzed, we found significant reduction in the risk of dying in the hospital after major or minor LEAs. Possible reasons for the decrease overtime in IHM after LEAs have been suggested by several authors [48,49,50,51]. The mortality reductions might indicate a more aggressive treatment of comorbidities prior to surgery, and improvements in surgical and perioperative care, which include better management of postoperative complications. Furthermore, the development of a critical pathway approach, multidisciplinary teams and surgical centers specializing in highly specific management of very high risk patients may have also contributed to lower mortality rates [50,51].

A remarkable result of our investigation is that among women and men with T2DM who underwent a LEAs, the prevalence of cardiovascular risk factors and chronic conditions has increased overtime. A recent study conducted in Spain that analyzed the changes in the health profile of people with T2DM from 2007 to 2018 agreed with us, finding an increment in the prevalence of hypertension, dyslipidemia, heart failure, and chronic kidney disease [52]. Suggested explanations for this increase include the aging population, improvement in registration, lowering the limit for hypercholesterolemia diagnosis and the improvement in the quality of care for people with T2DM, which would result in earlier detection of risk factors and chronic conditions and therefore reduce the prevalence [52,53].

The strengths of our study include its high external validity, as we analyzed records from almost all Spanish hospitals for an 18-year period, and the previously reported validity of T2DM and LEA coding in discharge records [3,4,36,54]. 

Limitations that have to be considered are those derived from the use of administrative databases, such as the lack of data on T2DM characteristics (duration, treatments or glycaemic control), the severity and treatment of concomitant conditions and the impossibility of detecting those patients who had been admitted to different hospitals the same year. Finally, as commented in the methods section, we excluded the year 2016 because the Spanish Ministry of Health made an informative note reporting that some autonomous regions in Spain had not sent their data for that year, due to the change from ICD9 to ICD10 [11,12]. It was estimated that around 5% of hospital admissions were not reported that year, with an unequal geographical distribution. When the statistics for year 2016 were run, we confirmed that some regions had not provided data, and we considered that this could bias our results, so we decided to exclude that year. However, we believe that this limitation would possibly have had very little effect on our main conclusions.

## 5. Conclusions

In conclusion, in Spain from 2001 to 2019, our data showed major sex differences, indicating decreasing and increasing LEA trends among men and women respectively; furthermore, women presented significantly higher IHM after minor and major LEA procedures than men. Older age and comorbidities, especially heart failure, are associated with higher IHM in men and women with T2DM following minor and major LEA procedures. 

## Figures and Tables

**Table 1:** Hospital discharges after minor lower-extremity amputations in Spain among people with T2DM from 2001–2019, according to sex.

Variable	Sex	2001–2003	2004–2006	2007–2009	2010–2012	2013–2015	2017–2019	Total	Trend
N (Inc per 100,000)	Men	6926 (254.77)	8376 (306.09)	9618 (338.24)	10,633 (351.85)	11,593 (362.22)	12,642 (381.49)	59,788 (335.23)	<0.001
	Women	2661 (83.49)	2850 (89.34)	2935 (91.54)	3082 (95.31)	2915 (89.4)	2851 (85.61)	17,294 (89.11)	0.326
Age, Mean (SD)	Men	67.17 (10.65)	66.89 (11)	67.32 (11.13)	67.22 (11.23)	67.31 (11.19)	67.81 (10.95)	67.32 (11.05)	<0.001
	Women	71.81 (10.75)	71.91 (11.07)	72 (11.89)	72.52 (11.73)	73.23 (12.02)	72.48 (12.27)	72.33 (11.66)	<0.001
CCI, Mean (SD)	Men	0.97 (0.86)	1.04 (0.9)	1.11 (0.93)	1.17 (0.95)	1.23 (0.96)	1.25 (1.07)	1.14 (0.96)	<0.001
	Women	0.84 (0.77)	0.96 (0.88)	1.01 (0.88)	1.09 (0.89)	1.18 (0.91)	1.19 (1.03)	1.05 (0.91)	<0.001
PVD, *n* (%)	Men	4085 (58.98)	4935 (58.92)	5840 (60.72)	6512 (61.24)	7638 (65.88)	6915 (54.7)	35,925 (60.09)	0.103
	Women	1412 (53.06)	1555 (54.56)	1639 (55.84)	1843 (59.8)	1809 (62.06)	1477 (51.81)	9735 (56.29)	0.010
IHD, *n* (%)	Men	966 (13.95)	1283 (15.32)	1623 (16.87)	1966 (18.49)	2089 (18.02)	2566 (20.3)	10,493 (17.55)	<0.001
	Women	303 (11.39)	404 (14.18)	432 (14.72)	429 (13.92)	393 (13.48)	405 (14.21)	2366 (13.68)	0.051
CKD, *n* (%)	Men	608 (8.78)	1008 (12.03)	1328 (13.81)	1850 (17.4)	2288 (19.74)	2994 (23.68)	10,076 (16.85)	<0.001
	Women	216 (8.12)	297 (10.42)	407 (13.87)	500 (16.22)	611 (20.96)	747 (26.2)	2778 (16.06)	<0.001
Hypertension, *n* (%)	Men	2128 (30.72)	3284 (39.21)	4241 (44.09)	5013 (47.15)	5615 (48.43)	6101 (48.26)	26,382 (44.13)	<0.001
	Women	1110 (41.71)	1374 (48.21)	1596 (54.38)	1721 (55.84)	1579 (54.17)	1462 (51.28)	8842 (51.13)	<0.001
Stroke, *n* (%)	Men	287 (4.14)	383 (4.57)	489 (5.08)	567 (5.33)	576 (4.97)	574 (4.54)	2876 (4.81)	0.242
	Women	104 (3.91)	153 (5.37)	124 (4.22)	131 (4.25)	139 (4.77)	126 (4.42)	777 (4.49)	0.131
HF, *n* (%)	Men	332 (4.79)	420 (5.01)	630 (6.55)	806 (7.58)	867 (7.48)	1283 (10.15)	4338 (7.26)	<0.001
	Women	167 (6.28)	209 (7.33)	247 (8.42)	252 (8.18)	287 (9.85)	299 (10.49)	1461 (8.45)	<0.001
Lipid disorders, *n* (%)	Men	722 (10.42)	1415 (16.89)	2320 (24.12)	3406 (32.03)	4463 (38.5)	5808 (45.94)	18,134 (30.33)	<0.001
	Women	296 (11.12)	514 (18.04)	745 (25.38)	972 (31.54)	1079 (37.02)	1310 (45.95)	4916 (28.43)	<0.001
LOHS, Median (IQR)	Men	18 (20)	17 (19)	17 (20)	15 (18)	14 (17)	14 (16)	15 (19)	<0.001
	Women	17 (18)	16 (18)	16 (18)	14 (17)	14 (16)	13 (17)	15 (18)	<0.001
IHM, N (%)	Men	186 (2.69)	194 (2.32)	248 (2.58)	224 (2.11)	195 (1.68)	237 (1.87)	1284 (2.15)	<0.001
	Women	106 (3.98)	111 (3.89)	121 (4.12)	98 (3.18)	109 (3.74)	78 (2.74)	623 (3.6)	<0.001

N: Number of procedures; Inc: Incidence per 100.000 T2DM; CCI (Charlson Comorbidity Index): Comorbidities included in the Charlson comorbidity index, except diabetes; PVD: Peripheral Vascular Disease; IHD: Ischemic Heart Disease; CKD: Chronic Kidney Disease; HF: Heart Failure; LOHS: Length of hospital stay; IHM: In-hospital mortality.

**Table 2:** Hospital discharges after major lower-extremity amputations in Spain among people with T2DM in 2001–2019, according to sex.

	Sex	2001–2003	2004–2006	2007–2009	2010–2012	2013–2015	2017–2019	Total	Trend
N (Inc per 100,000)	Men	4715 (173.44)	5589 (204.24)	6111 (214.91)	5951 (196.92)	5925 (185.12)	5926 (178.82)	34,217 (191.85)	0.004
	Women	3223 (101.12)	3480 (109.09)	3302 (102.98)	2946 (91.11)	2580 (79.13)	2229 (66.93)	17,760 (91.51)	<0.001
Age, Mean (SD)	Men	71.34 (10)	71.51 (10.22)	71.58 (10.41)	71.82 (10.62)	71.64 (10.67)	71.76 (10.47)	71.62 (10.42)	0.190
	Women	76.65 (9.15)	77.09 (9.09)	77.69 (9.59)	77.89 (10.07)	77.68 (10.35)	77.09 (10.73)	77.34 (9.77)	<0.001
CCI, Mean (SD)	Men	1.31 (0.93)	1.41 (0.98)	1.44 (0.98)	1.5 (1)	1.55 (0.99)	1.66 (1.13)	1.48 (1.01)	<0.001
	Women	1.17 (0.87)	1.26 (0.89)	1.28 (0.92)	1.38 (0.95)	1.44 (0.93)	1.52 (1.02)	1.33 (0.93)	<0.001
PVD, *n* (%)	Men	3153 (66.87)	3862 (69.1)	4278 (70)	4201 (70.59)	4388 (74.06)	3976 (67.09)	23,858 (69.73)	0.005
	Women	2022 (62.74)	2299 (66.06)	2138 (64.75)	2025 (68.74)	1830 (70.93)	1449 (65.01)	11,763 (66.23)	<0.001
IHD, *n* (%)	Men	838 (17.77)	1127 (20.16)	1223 (20.01)	1323 (22.23)	1314 (22.18)	1404 (23.69)	7229 (21.13)	<0.001
	Women	526 (16.32)	625 (17.96)	582 (17.63)	498 (16.9)	466 (18.06)	401 (17.99)	3098 (17.44)	0.382
CKD, *n* (%)	Men	607 (12.87)	890 (15.92)	1151 (18.83)	1275 (21.42)	1480 (24.98)	1777 (29.99)	7180 (20.98)	<0.001
	Women	344 (10.67)	453 (13.02)	524 (15.87)	571 (19.38)	602 (23.33)	662 (29.7)	3156 (17.77)	<0.001
Hypertension, *n* (%)	Men	1548 (32.83)	2403 (43)	2877 (47.08)	2765 (46.46)	2828 (47.73)	2730 (46.07)	15151 (44.28)	<0.001
	Women	1408 (43.69)	1831 (52.61)	1946 (58.93)	1636 (55.53)	1381 (53.53)	1116 (50.07)	9318 (52.47)	<0.001
Stroke, *n* (%)	Men	435 (9.23)	546 (9.77)	622 (10.18)	564 (9.48)	577 (9.74)	573 (9.67)	3317 (9.69)	0.760
	Women	301 (9.34)	342 (9.83)	321 (9.72)	305 (10.35)	254 (9.84)	173 (7.76)	1696 (9.55)	0.046
HF, *n* (%)	Men	383 (8.12)	548 (9.8)	597 (9.77)	714 (12)	679 (11.46)	930 (15.69)	3851 (11.25)	<0.001
	Women	331 (10.27)	377 (10.83)	400 (12.11)	382 (12.97)	283 (10.97)	334 (14.98)	2107 (11.86)	<0.001
Lipid disorders, *n* (%)	Men	434 (9.2)	912 (16.32)	1351 (22.11)	1815 (30.5)	2187 (36.91)	2722 (45.93)	9421 (27.53)	<0.001
	Women	296 (9.18)	569 (16.35)	744 (22.53)	900 (30.55)	982 (38.06)	1009 (45.27)	4500 (25.34)	<0.001
LOHS, Median (IQR)	Men	20 (21)	19 (21)	18 (22)	16 (20)	15 (18)	15 (19)	17 (20)	<0.001
	Women	17 (19)	16 (18)	15 (18)	14 (16)	14 (15)	13 (15)	15 (17)	<0.001
IHM, N (%)	Men	465 (9.86)	529 (9.47)	554 (9.07)	507 (8.52)	482 (8.14)	474 (8)	3011 (8.8)	0.002
	Women	358 (11.11)	395 (11.35)	434 (13.14)	344 (11.68)	291 (11.28)	226 (10.14)	2048 (11.53)	0.019

N: Number of procedures; Inc: Incidence per 100.000 T2DM; CCI (Charlson Comorbidity Index): Comorbidities included in the Charlson comorbidity index, except diabetes; PVD: Peripheral Vascular Disease; IHD: Ischemic Heart Disease; CKD: Chronic Kidney Disease; HF: Heart Failure; LOHS: Length of hospital stay; IHM: In-hospital mortality.

**Table 3:** In-hospital mortality for T2DM subjects hospitalized in Spain from 2001 to 2019 with non-traumatic lower-extremity amputations according to sex.

		Minor LEAs		Major LEAs	
		Men *n* (%)	Women *n* (%)	Men *n* (%)	Women *n* (%)
Age groups (Years) ^a,b,c,d^	18–49	8 (0.24)	4 (0.53)	36 (4.05)	12 (5.08)
	50–59	72 (0.6)	21 (1.16)	196 (5.14)	63 (8.24)
	60–69	252 (1.41)	50 (1.42)	615 (7.18)	206 (9.22)
	70–79	487 (2.71)	199 (3.3)	1147 (9.13)	638 (10.24)
	≥80	465 (5.35)	349 (6.74)	1017 (12.13)	1129 (13.61)
PVD ^a,b,c,d^	Presence	891 (2.48)	372 (3.82)	2019 (8.46)	1235 (10.5)
IHD ^a,b,c,d^	Presence	405 (3.86)	151 (6.38)	798 (11.04)	451 (14.56)
CKD ^a,b,c,d^	Presence	415 (4.12)	165 (5.94)	928 (12.92)	513 (16.25)
Hypertension ^a,b,c,d^	Presence	419 (1.59)	231 (2.61)	1055 (6.96)	924 (9.92)
Stroke ^a,b,c,d^	Presence	135 (4.69)	57 (7.34)	380 (11.46)	254 (14.98)
HF ^a,b,c,d^	Presence	445 (10.26)	205 (14.03)	763 (19.81)	544 (25.82)
Lipid disorders ^a,b,c,d^	Presence	261 (1.44)	132 (2.69)	657 (6.97)	391 (8.69)
Year ^a,b,c,d^	2001–2003	186 (2.69)	106 (3.98)	465 (9.86)	358 (11.11)
	2004–2006	194 (2.32)	111 (3.89)	529 (9.47)	395 (11.35)
	2007–2009	248 (2.58)	121 (4.12)	554 (9.07)	434 (13.14)
	2010–2012	224 (2.11)	98 (3.18)	507 (8.52)	344 (11.68)
	2013–2015	195 (1.68)	109 (3.74)	482 (8.14)	291 (11.28)
	2017–2019	237 (1.87)	78 (2.74)	474 (8)	226 (10.14)

PVD: Peripheral Vascular Disease; IHD: Ischemic Heart Disease; CKD: Chronic Kidney Disease; HF: Heart Failure ^a^ Significant association of the study variable with in-hospital mortality among men with T2DM after minor non-traumatic lower-extremity amputation. ^b^ Significant association of the study variable with in-hospital mortality among women with T2DM after minor non-traumatic lower-extremity amputation. ^c^ Significant association of the study variable with in-hospital mortality among men with T2DM after major non-traumatic lower-extremity amputation. ^d^ Significant association of the study variable with in-hospital mortality among women with T2DM after major non-traumatic lower-extremity amputation.

**Table 4:** Multivariable analysis of the factors associated with in-hospital mortality for T2DM subjects hospitalized in Spain from 2001 to 2019 with non-traumatic lower-extremity amputations.

		Minor OR (95%CI)	Major OR (95%CI)
Age groups (Years)	18–49	1	1
	50–59	2.27 (1.24–4.15)	1.3 (0.95–1.79)
	60–69	4.17 (2.33–7.44)	1.72 (1.27–2.32)
	70–79	7.52 (4.24–13.35)	2.18 (1.62–2.93)
	≥80	15.6 (8.79–27.7)	3.15 (2.35–4.24)
IHD	Presence	1.61 (1.45–1.8)	1.29 (1.2–1.38)
CKD	Presence	1.6 (1.43–1.79)	1.58 (1.47–1.7)
Stroke	Presence	2.06 (1.76–2.42)	1.46 (1.33–1.6)
HF	Presence	5.11 (4.61–5.68)	2.91 (2.71–3.13)
Year	2001–2003	1	1
	2004–2006	0.87 (0.73–1.03)	0.98 (0.89–1.09)
	2007–2009	0.9 (0.77–1.06)	1.02 (0.92–1.13)
	2010–2012	0.69 (0.58–0.82)	0.89 (0.8–0.99)
	2013–2015	0.6 (0.51–0.71)	0.87 (0.78–0.97)
	2017–2019	0.54 (0.45–0.64)	0.75 (0.67–0.84)
Sex	Women	1.3 (1.17–1.44)	1.18 (1.11–1.26)

IHD: Ischemic Heart Disease; CKD: Chronic Kidney Disease; HF: Heart Failure; OR. Odds Ratio. Calculated using logistic regression models: Odds Ratio (OR). The logistic regression multivariable models were built using “death (yes/no)” as dependent variables.

## Data Availability

According to the contract signed with the Spanish Ministry of Health and Social Services, which provided access to the databases from the Spanish National Hospital Database (RAE-CMBD, Registro de Actividad de Atención Especializada. Conjunto Mínimo Básico de Datos, Registry of Specialized Health Care Activities. Minimum Basic Data Set), we cannot share the databases with any other investigator, and we have to destroy the databases once the investigation has concluded. Consequently, we cannot upload the databases to any public repository. However, any investigator can apply for access to the databases by filling out the questionnaire available at http://www.msssi.gob.es/estadEstudios/estadisticas/estadisticas/estMinisterio/SolicitudCMBDdocs/Formulario_Peticion_Datos_CMBD.pdf (accessed on 4 January 2022). All other relevant data are included in the paper.

## Supplementary Materials

The following are available online at https://www.mdpi.com/article/10.3390/jcm11051246/s1, Table S1. International Classification of Disease, 9th edition (ICD-9-CM) and 10th edition, (ICD-10) codes for the clinical diagnoses and procedures used in this investigation. Table S2. Multivariable analysis of the factors associated with in-hospital mortality for men and women with T2DM hospitalized in Spain from 2001 to 2019 with non-traumatic lower-extremity amputations.

## References

[1] Stern J.R., Wong C.K., Yerovinkina M., Spindler S.J., See A.S., Panjaki S., Loven S.L., D’Andrea R.F., Nowygrod R. (2017). A Meta-analysis of long-term mortality and associated risk factors following lower extremity amputation. Ann. Vasc. Surg..

[2] López-De-Andrés A., Martínez-Huedo M.A., Carrasco-Garrido P., Hernández-Barrera V., Gil-de-Miguel A., Jiménez-García R. (2011). Trends in lower-extremity amputations in people with and without diabetes in Spain, 2001–2008. Diabetes Care.

[3] Lopez-De-Andres A., Jiménez-García R., Aragón-Sánchez J., Jiménez-Trujillo I., Hernández-Barrera V., Méndez-Bailón M., de Miguel-Yanes J.M., Perez-Farinos N., Carrasco-Garrido P. (2015). National trends in incidence and outcomes in lower extremity amputations in people with and without diabetes in Spain, 2001–2012. Diabetes Res. Clin. Pract..

[4] Carinci F., Uccioli L., Benedetti M.M., Klazinga N.S. (2020). An in-depth assessment of diabetes-related lower extremity amputation rates 2000–2013 delivered by twenty-one countries for the data collection 2015 of the Organization for Economic Cooperation and Development (OECD). Acta Diabetologica.

[5] Harding J.L., Pavkov M.E., Gregg E.W., Burrows N.R. (2019). Trends of nontraumatic lower-extremity amputation in end-stage renal disease and diabetes: United States, 2000–2015. Diabetes Care.

[6] Icks A., Haastert B., Trautner C., Giani G., Glaeske G., Hoffmann F. (2009). Incidence of lower-limb amputations in the diabetic compared to the non-diabetic population. findings from nationwide insurance data, Germany, 2005–2007. Exp. Clin. Endocrinol. Diabetes.

[7] Lombardo F.L., Maggini M., De Bellis A., Seghieri G., Anichini R. (2014). Lower extremity amputations in persons with and without diabetes in Italy: 2001–2010. PLoS ONE.

[8] Kamitani F., Nishioka Y., Noda T., Myojin T., Kubo S., Higashino T., Okada S., Akai Y., Ishii H., Takahashi Y. (2021). Incidence of lower limb amputation in people with and without diabetes: A nationwide 5-year cohort study in Japan. BMJ Open.

[9] Chahrour M.A., Habib J.R., El Moheb M.N., Cherfan P., Mahmoud D., El Rahyel A., Khachfe H., Hoballah J.J. (2021). Incidence and predictors of surgical site infection complications in diabetic patients undergoing lower limb amputation. Ann. Vasc. Surg..

[10] Ministerio de Sanidad, Consumo y Bienestar Social Registro de Actividad de Atención Especializada. RAE-CMBD. https://www.mscbs.gob.es/estadEstudios/estadisticas/cmbdhome.htm.

[11] Gogorcena M.A. (2017). The adoption of the ICD-10-ES codification rules or how to make of necessity a virtue. Rev. Calid. Asist..

[12] Ministerio de Sanidad, Consumo y Bienestar Social (2021). Atención Perinatal en España: Análisis de los Recursos Físicos, Humanos, Actividad y Calidad de los Servicios Hospitalarios, 2010–2018.

[13] Soriguer F., Goday A., Bosch-Comas A., Bordiú E., Calle-Pascual A., Carmena R., Casamitjana R., Castaño L., Castell C., Catalá M. (2012). Prevalence of diabetes mellitus and impaired glucose regulation in Spain: The Di@bet.es Study. Diabetologia.

[14] Ministerio de Sanidad, Consumo y Bienestar Social Encuesta Nacional de Salud de España. https://www.mscbs.gob.es/estadEstudios/estadisticas/encuestaNacional/.

[15] Sundararajan V., Henderson T., Perry C., Muggivan A., Quan H., Ghali W.A. (2004). New ICD-10 version of the Charlson comorbidity index predicted in-hospital mortality. J. Clin. Epidemiol..

[16] Quan H., Sundararajan V., Halfon P., Fong A., Burnand B., Luthi J.-C., Saunders L.D., Beck C.A., Feasby T.E., Ghali W.A. (2005). Coding algorithms for defining comorbidities in ICD-9-CM and ICD-10 administrative data. Med. Care.

[17] Kim H.J., Fay M.P., Feuer E.J., Midthune D.N. (2000). Permutation tests for join-point regression with applications to cancer rates. Stat. Med..

[18] Ministerio de Sanidad, Consumo y Bienestar Social Solicitud de Extracción de Datos—Extraction Request (Spanish National Hospital Discharge Database). https://www.mscbs.gob.es/estadEstudios/estadisticas/estadisticas/estMinisterio/SolicitudCMBDdocs/2018_Formulario_Peticion_Datos_RAE_CMBD.pdf.

[19] Wu H., Yang A., Lau E.S.H., Ma R.C.W., Kong A.P.S., Chow E., So W.-Y., Chan J.C.N., Luk A.O.Y. (2020). Secular trends in rates of hospitalisation for lower extremity amputation and 1 year mortality in people with diabetes in Hong Kong, 2001–2016: A retrospective cohort study. Diabetologia.

[20] Iacopi E., Pieruzzi L., Riitano N., Abbruzzese L., Goretti C., Piaggesi A. (2021). The weakness of the strong sex: Differences between men and women affected by diabetic foot disease. Int. J. Low. Extrem. Wounds.

[21] Déruaz-Luyet A., Ms C.R., Garry E.M., Brodovicz K.G., Lavery L.A. (2020). Incidence of lower extremity amputations among patients with type 1 and type 2 diabetes in the United States from 2010 to 2014. Diabetes Obes. Metab..

[22] Tivesten A., Mellström D., Jutberger H., Fagerberg B., Lernfelt B., Orwoll E., Karlsson M.K., Ljunggren O., Ohlsson C. (2007). Low serum testosterone and high serum estradiol associate with lower extremity peripheral arterial disease in elderly men. The MrOS study in Sweden. J. Am. Coll. Cardiol..

[23] Peek M.E. (2011). Gender differences in diabetes-related lower extremity amputations. Clin. Orthop. Relat. Res..

[24] Ferranti K.M., Osler T.M., Duffy R.P., Stanley A.C., Bertges D.J. (2015). Association between gender and outcomes of lower extremity peripheral vascular interventions. J. Vasc. Surg..

[25] Shin J.Y., Roh S.-G., Lee N.-H., Yang K.-M. (2017). Influence of epidemiologic and patient behavior-related predictors on amputation rates in diabetic patients: Systematic review and meta-analysis. Int. J. Low. Extrem. Wounds.

[26] Fan L., Wu X.J. (2021). Sex difference for the risk of amputation in diabetic patients: A systematic review and meta-analysis. PLoS ONE.

[27] Wong M.W. (2006). Predictors for mortality after lower-extremity amputations in geriatric patients. Am. J. Surg..

[28] Gurney J.K., Stanley J., Rumball-Smith J., York S., Sarfati D. (2018). Postoperative death after lower-limb amputation in a national prevalent cohort of patients with diabetes. Diabetes Care.

[29] Deery S.E., Soden P.A., Zettervall S.L., Shean K.E., Bodewes T.C.F., Pothof A.B., Lo R.C., Schermerhorn M.L. (2017). Sex differences in mortality and morbidity following repair of intact abdominal aortic aneurysms. J. Vasc. Surg..

[30] Kautzky-Willer A., Harreiter J., Pacini G. (2016). Sex and gender differences in risk, pathophysiology and complications of type 2 diabetes mellitus. Endocr. Rev..

[31] Seghieri G., Policardo L., Anichini R., Franconi F., Campesi I., Cherchi S., Tonolo G. (2017). The effect of sex and gender on diabetic complications. Curr. Diabetes Rev..

[32] Rossi M.C., Cristofaro M.R., Gentile S., Lucisano G., Manicardi V., Mulas M.F., Napoli A., Nicolucci A., Pellegrini F., Suraci C. (2013). Sex disparities in the quality of diabetes care: Biological and cultural factors may play a different role for different outcomes: A cross-sectional observational study from the AMD annals initiative. Diabetes Care.

[33] Franzini L., Ardigò D., Cavalot F., Miccoli R., Rivellese A., Trovati M., Zavaroni I., Vaccaro O. (2013). Women show worse control of type 2 diabetes and cardiovascular disease risk factors than men: Results from the MIND.IT Study Group of the Italian Society of Diabetology. Nutr. Metab. Cardiovasc. Dis..

[34] Wannamethee S.G., Papacosta O., Lawlor A.D., Whincup P., Lowe G.D., Ebrahim S., Sattar N. (2012). Do women exhibit greater differences in established and novel risk factors between diabetes and non-diabetes than men? The British regional heart study and British women’s heart health study. Diabetologia.

[35] Policardo L., Seghieri G., Francesconi P., Anichini R., Franconi F., Del Prato S. (2017). Gender difference in diabetes related excess risk of cardiovascular events: When does the ‘risk window’ open?. J. Diabetes Complicat..

[36] De Miguel-Yanes J.M., Garcia R.J., Hernández-Barrera V., Méndez-Bailón M., de Miguel-Díez J., Lopez-De-Andrés A. (2017). Impact of type 2 diabetes mellitus on in-hospital-mortality after major cardiovascular events in Spain (2002–2014). Cardiovasc. Diabetol..

[37] Claessen H., Avalosse H., Guillaume J., Narres M., Kvitkina T., Arend W., Morbach S., Lauwers P., Nobels F., Boly J. (2018). Decreasing rates of major lower-extremity amputation in people with diabetes but not in those without: A nationwide study in Belgium. Diabetologia.

[38] Ahmad N., Thomas G.N., Gill P., Torella F. (2016). The prevalence of major lower limb amputation in the diabetic and non-diabetic population of England 2003–2013. Diab. Vasc. Dis. Res..

[39] Rasmussen B.S.B., Yderstraede K., Carstensen B., Skov O., Beck-Nielsen H. (2016). Substantial reduction in the number of amputations among patients with diabetes: A cohort study over 16 years. Diabetologia.

[40] Geiss L.S., Li Y., Hora I., Albright A., Rolka D., Gregg E.W. (2019). Resurgence of diabetes-related nontraumatic lower-extremity amputation in the young and middle-aged adult, U.S. population. Diabetes Care.

[41] Hussain M.A., Al-Omran M., Salata K., Sivaswamy A., Forbes T.L., Sattar N., Aljabri B., Kayssi A., Verma S., de Mestral C. (2019). Population-based secular trends in lower-extremity amputation for diabetes and peripheral artery disease. Can. Med. Assoc. J..

[42] Schofield C.J., Yu N., Jain A.S., Leese G.P. (2009). Decreasing amputation rates in patients with diabetes—A population-based study. Diabet. Med..

[43] Ploeg A., Lardenoye J.-W., Peeters M.-P.V., Breslau P.J. (2005). Contemporary series of morbidity and mortality after lower limb amputation. Eur. J. Vasc. Endovasc. Surg..

[44] Jones W.S., Patel M.R., Dai D., Vemulapalli S., Subherwal S., Stafford J., Peterson E.D. (2013). High mortality risks after major lower extremity amputation in Medicare patients with peripheral artery disease. Am. Heart J..

[45] Shah S.K., Bena J.F., Allemang M.T., Kelso R., Clair D.G., Vargas L., Kashyap V.S. (2013). Lower extremity amputations: Factors associated with mortality or contralateral amputation. Vasc. Endovascular. Surg..

[46] Beyaz S., Guler U.O., Bağır G.Ş. (2017). Factors affecting lifespan following below-knee amputation in diabetic patients. Acta Orthop. Traumatol. Turc..

[47] Andersen J.C., Mannoia K.A., Patel S.T., Leong B.V., Murga A.G., Teruya T.H., Kiang S.C., Abou-Zamzam A.M. (2021). Factors affecting one-year outcomes after major lower extremity amputation in the vascular quality initiative amputation registry. Am. Surg..

[48] Spoden M., Nimptsch U., Mansky T. (2019). Amputation rates of the lower limb by amputation level—Observational study using German national hospital discharge data from 2005 to 2015. BMC Health Serv. Res..

[49] López-Valverde M.E., Aragón-Sánchez J., López-De-Andrés A., Guerrero-Cedeño V., Tejedor-Méndez R., Víquez-Molina G., Jiménez-García R. (2018). Perioperative and long-term all-cause mortality in patients with diabetes who underwent a lower extremity amputation. Diabetes Res. Clin. Pract..

[50] Martínez-Gómez D.A., Moreno-Carrillo M.A., Campillo-Soto A., Carrillo-García A., Aguayo-Albasini J.L. (2014). Reduction in diabetic amputations over 15 years in a defined Spain population. Benefits of a critical pathway approach and multidisciplinary team work. Rev. Esp. Quimioter..

[51] Rubio J.A., Aragón-Sánchez J., Jiménez S., Guadalix G., Albarracín A., Salido C., Sanz-Moreno J., Ruiz-Grande F., Gil-Fournier N., Álvarez J. (2014). Reducing major lower extremity amputations after the introduction of a multidisciplinary team for the diabetic foot. Int. J. Low. Extrem. Wounds.

[52] Mata-Cases M., Vlacho B., Real J., Puig-Treserra R., Bundó M., Franch-Nadal J., Mauricio D. (2022). Trends in the degree of control and treatment of cardiovascular risk factors in people with type 2 diabetes in a primary care setting in Catalonia during 2007–2018. Front. Endocrinol..

[53] Guallar-Castillón P., Gil-Montero M., León-Muñoz L.M., Graciani A., Bayán-Bravo A., Taboada J.M., Banegas J.R., Rodríguez-Artalejo F. (2012). Magnitude and management of hypercholesterolemia in the adult population of Spain, 2008–2010: The ENRICA study. Rev. Esp. Cardiol..

[54] Buckley C.M., Kearney P.M., Ali F., Ni Bhuachalla C., Casey C., Roberts G., Perry I.J., Bradley C.P. (2013). Concordance studies between hospital discharge data and medical records for the recording of lower extremity amputation and diabetes in the Republic of Ireland. BMC Res. Notes.

