# Supplementary material for: Trends of Non-Traumatic Lower-Extremity Amputation and Type 2 Diabetes: Spain, 2001–2019
Source: J Clin Med. 2022 Feb 25;11(5):1246. doi: 10.3390/jcm11051246 (PMC8911304; doi:10.3390/jcm11051246)
Supplement: Supplementary file 1 [file jcm-11-01246-s001.zip › jcm-1581531-supplementary.pdf]

Supplementary Table S1. International Classification of Disease, 9th edition (ICD-9-CM) and 10th edition, (ICD-10) codes for the clinical diagnoses and procedures used in this investigation.

	ICD-9 code	ICD-10 code
**Type 2 diabetes**	250.x0; 250.x2	E11.xxx
**Type 1 diabetes (exclusion)**	250.x1; 250.x3.	E10.xxx
**Level of NLEA**		
Toe	84.11	0Y6P – 0Y6Y
Foot	84.12, 84.13	0Y6M – 0Y6N
Below the knee amputation (BKA)	84.14, 84.15, 84.16	0Y6F, 0Y6G, 0Y6H, 0YJ
Above the knee amputation (AKA)	84.17, 84.18, 84.19	0Y62, 0Y63, 0Y64, 0Y67, 0Y68, 0Y6C, 0Y6D
Traumatic amputations (exclusion)	895, 896, 897	S78, S88, S98
**Peripheral vascular disease**	0.93.0,473.3,440.x,441.x,443.1-443.9,447.1,557.1,557.9,V43.4	I70.x, I71.x, I73.1, I73.8, I73.9, I77.1, I79.0, I79.2, K55.1, K55.8, K55.9, Z95.8, Z95.9
**Ischemic heart disease**	410-414	I20-I25
**Chronic kidney disease**	585	N18
**Hypertension**	401.1, 401.9, 642.0	I10, I16.6
**Stroke**	433.xx, 434,xx, 436	I60-I66
**Heart failure**	398.91,402.01,402.11,402.91,404.01, 404.03,404.11,404.13,404.91,404.93,425.4–425.9, 428.x	I09.9, I11.0, I13.0, I13.2, I25.5, I42.0, I42.5–I42.9, I43.x, I50.x, P29.0
**Lipid metabolism disease**	272,0-272.4, 272.8, 272.9	E78.0X-E78.5

Supplementary Table S2. Multivariate analysis of the factors associated with in-hospital mortality for men and women with T2DM hospitalized in Spain from 2001 to 2019 with nontraumatic lower extremity amputations

		MEN		WOMEN	
		**Minor**	**Major**	**Minor**	**Major**
Age groups (Years)	18-49	1	1	1	1
	50-59	2.41(1.16-5.02)	1.25(0.86-1.79)	2.13(0.73-6.25)	1.62(0.85-3.07)
	60-69	4.98(2.45-10.1)	1.72(1.22-2.43)	2.33(0.84-6.49)	1.76(0.96-3.22)
	70-79	8.62(4.27-17.41)	2.26(1.61-3.18)	5.11(1.89-13.85)	2.02(1.12-3.66)
	≥80	17.15(8.49-34.66)	3.22(2.29-4.54)	11.41(4.23-30.81)	2.99(1.66-5.4)
IHD	Presence	1.56(1.38-1.78)	1.27(1.16-1.39)	1.73(1.41-2.11)	1.33(1.19-1.5)
CKD	Presence	1.65(1.45-1.89)	1.58(1.44-1.73)	1.47(1.19-1.81)	1.58(1.39-1.78)
Stroke	Presence	2.08(1.72-2.52)	1.43(1.27-1.61)	2.02(1.5-2.72)	1.5(1.29-1.73)
HF	Presence	5.41(4.76-6.14)	2.84(2.59-3.12)	4.55(3.78-5.48)	3.03(2.7-3.4)
Year	2001-03	1	1	1	1
	2004-06	0.86(0.69-1.06)	0.94(0.82-1.08)	0.89(0.67-1.18)	1.04(0.89-1.21)
	2007-09	0.87(0.71-1.07)	0.9(0.79-1.03)	0.96(0.73-1.27)	1.2(1.03-1.41)
	2010-12	0.68(0.56-0.84)	0.81(0.7-0.93)	0.71(0.53-0.95)	1.02(0.87-1.21)
	2013-15	0.54(0.44-0.67)	0.78(0.68-0.9)	0.74(0.55-0.99)	1.02(0.86-1.22)
	2017-19	0.54(0.44-0.67)	0.69(0.6-0.8)	0.5(0.36-0.7)	0.83(0.69-1)

IHD: Ischemic Heart Disease; CKD: Chronic Kidney Disease; HF: Heart Failure.
OR. Odds Ratio. Calculated using logistic regression models: Odds Ratio (OR). The logistic regression multivariable models were built using "death (yes/no)" as dependent variables
